# Choices and Challenges With Treatment of Myasthenia Gravis in Pregnancy: A Systematic Review

**DOI:** 10.7759/cureus.42772

**Published:** 2023-07-31

**Authors:** Lakshya Kumar, Meet Popatbhai Kachhadia, Jashanpreet Kaur, Harshkumar Patel, Khutaija Noor, Rushi G Gohel, Paramjeet Kaur, Siddharth Raiyani, Vatsal A Gohel, Advait M Vasavada

**Affiliations:** 1 Internal Medicine, Pandit Dindayal Upadhyay (PDU) Medical College, Rajkot, IND; 2 Internal Medicine, Mata Gujri Memorial Medical College, Kishanganj, IND; 3 Internal Medicine, Shadan Institute of Medical Sciences, Hyderabad, IND; 4 Internal Medicine, Guru Gobind Singh Medical College, Faridkot, IND; 5 Internal Medicine, Karaganda Medical University, Karaganda, KAZ; 6 Internal Medicine, M. P. Shah Medical College, Jamnagar, IND; 7 Internal Medicine, California Institute of Behavioral Neurosciences and Psychology, Fairfield, USA

**Keywords:** during pregnancy, pregnancy counseling, pregnancy, highrisk pregnancy, neuromuscular junction disorders, myasthenia gravis (mg) in pregnancy, myasthenia gravis (mg)

## Abstract

Myasthenia gravis (MG) is an autoimmune disease affecting young women in their second and third decades, coinciding with their reproductive years. We aim to explore the choices and challenges in the treatment of MG in pregnancy. Cochrane, PubMed, Google Scholar, and Embase were the four databases systematically searched for studies with patients reporting pregnancy outcomes for women with MG during pregnancy using the Preferred Reporting Items for Systematic Reviews and Meta-Analysis (PRISMA) technique. Quality assessment was done using the Joanna Briggs Institute critical tool (JBI, Adelaide, Australia) for methodological quality. From 2000 to 2023, 40 studies from database search results were considered. There is a substantial risk of complications with MG, especially if it appears during pregnancy. In particular, widespread weakness is a cause of severe, life-threatening disorders, but several treatment options are available.

## Introduction and background

Myasthenia gravis (MG) is one of the most commonly acquired neuromuscular disorders affecting almost one million globally [1,2]. MG is a form of autoimmune illness affecting neuromuscular transmission frequently related to autoantibodies acting on the nicotinic acetylcholine receptor (AChR) [3-5]. As a result of this event, there is a decrease in nerve impulse transmission to striated muscle fibers. Hyperplasia and thymic malignancies have been linked to aberrant autoantibody synthesis and secretion [4]. The skeletal muscles, particularly those of the respiratory, ocular, leg, and eye muscles, frequently experience varying weakening in the affected people [1]. The prevalence of MG is between one case per 100,000 people and one case per 50,000 people, with two-thirds of those affected being females [6]. While MG can strike at any age or stage of life, women are more likely than males to experience it, with cases often peaking in the third decade [7]. As a result, the reproductive stage of life is impacted, particularly during pregnancy [8].

The uncertain and unpredictable course of an episodic MG exacerbation necessitates more intensive medical attention [9,10]. In order to regulate their symptoms as their muscle weakness or other symptoms develop, those suffering an exacerbation may need to take more medication or other forms of medicine [11]. On the other hand, the myasthenic crisis is a potentially lethal illness characterized by the deterioration of the bulbar and respiratory muscles, which impairs breathing and necessitates a ventilator [12]. The diagnosis of MG is made through clinical and physical tests, and the diagnosis is confirmed through serum immunoassays that measure autoantibody levels [4]. The main goal of the available interventional MG treatments is to manage the disease's symptomatic stage by using anticholinesterase medications coupled with other immunosuppressive medications and steroids frequently used to treat other severe conditions [13,14]. The management of myasthenic crises and other refractory cases has also been supported by alternative therapies such as intravenous immunoglobulin (IVIg) and plasmapheresis [15].

Pregnancy-related MG can manifest itself in a number of different ways [10,16]. The aggravation of MG is known to occur throughout the first trimester of pregnancy and during the postpartum period, according to Tanacan et al. [17]. Although the illness can develop at any point in a person's life, Tanacan et al. noted that this is the case for most MG cases [17]. The high MG exacerbation rates have also been reported to be over 30% in pregnant women, whereas other investigations have found lower aggravation rates [18,19]. It is crucial to note that MG has a substantial influence on newborns in addition to pregnant mothers [20]. Both the mother and the newborn infant may have myasthenia symptoms in the case of maternal MG, such as varying degrees of weakness and skeletal muscle exhaustion [21].

In most cases, placentally transmitted antibodies to the AChR cause MG in the neonate (alias transient neonatal MG-TNMG), which impairs neuromuscular transmission [20,22,23]. It affects 10-15% of infants born to mothers with MG [22]. Following the dangers MG presents to women during pregnancy and consequential newborn issues, the current study systematically reviews the available literature to explore the effects of MG on women during pregnancy and the risks associated with pregnancy outcomes.

## Review

Materials and methods

Design and Literature Sources

The current study is a systematic review that adhered strictly to the Preferred Reporting Items for Systematic Reviews and Meta-Analyses (PRISMA) framework's suggested reporting criteria [24]. A thorough electronic database search was conducted to locate papers reporting on the impact of MG during pregnancy and the related effects on neonates. Google Scholar, Embase, Cochrane Library, and PubMed are among the databases that were searched for literature.

Search Strategy

The extensive database search was made possible thanks to the usage of the Boolean operators "AND" and "OR" along with keywords. The search was conducted using the terms "myasthenia gravis" OR "MG" OR "myasthenic crisis" AND "pregnancy" OR "pregnant women" OR "pregnancies" OR "obstetric delivery." Only English studies released between 2000 and 2023 were considered in the search. Recent research and quality of treatment after 2000 made it likely that a good quality of evidence was after that period.

Eligibility Criteria

Two independent reviewers were assigned to sort papers suitable to be considered for the current study based on predetermined eligibility criteria. The following inclusion criteria were used to acquire the included articles. Primary studies as well as review articles report the effect of MG in pregnancy and how pregnancy affects MG. Only free full-text papers available and done between 2000 and 2023 on MG in pregnancy were considered in the current study. Articles exploring MG without association with pregnancy, MG in men, other than women during pregnancy were ignored from inclusion. Similarly, papers in different languages done in 2000 were termed obsolete and excluded from the current study.

Data Extraction

Data extraction was then carried out by the two independent researchers using the population, intervention, control, and outcomes (PICO) framework [25] and the studies' established eligibility. The population was pregnant females, the intervention was pharmacotherapies and procedures, the comparison was standard of care, and outcome considerations were acute and chronic outcomes. Author information (name and year), study information (design, location, and period), participant information (sample size and characteristics), intervention information, and outcomes of interest were the information that was extracted.

Quality Appraisal

The quality of the included studies was evaluated using the Joanna Briggs Institute (JBI, Adelaide, Australia) tool for the methodological quality appraisal for retrospective case series [26]. The JBI Critical Appraisal Checklist for Case Series is a tool offered by JBI for evaluating case series research's level of quality. This instrument comprises nine criteria to assess the study's quality and applicability. A score is assigned to each criterion, which can be "yes," "no," "unclear," or "not applicable." We can then estimate the overall quality of the study as per the checklist [27].

Results and findings

**Figure 1:**
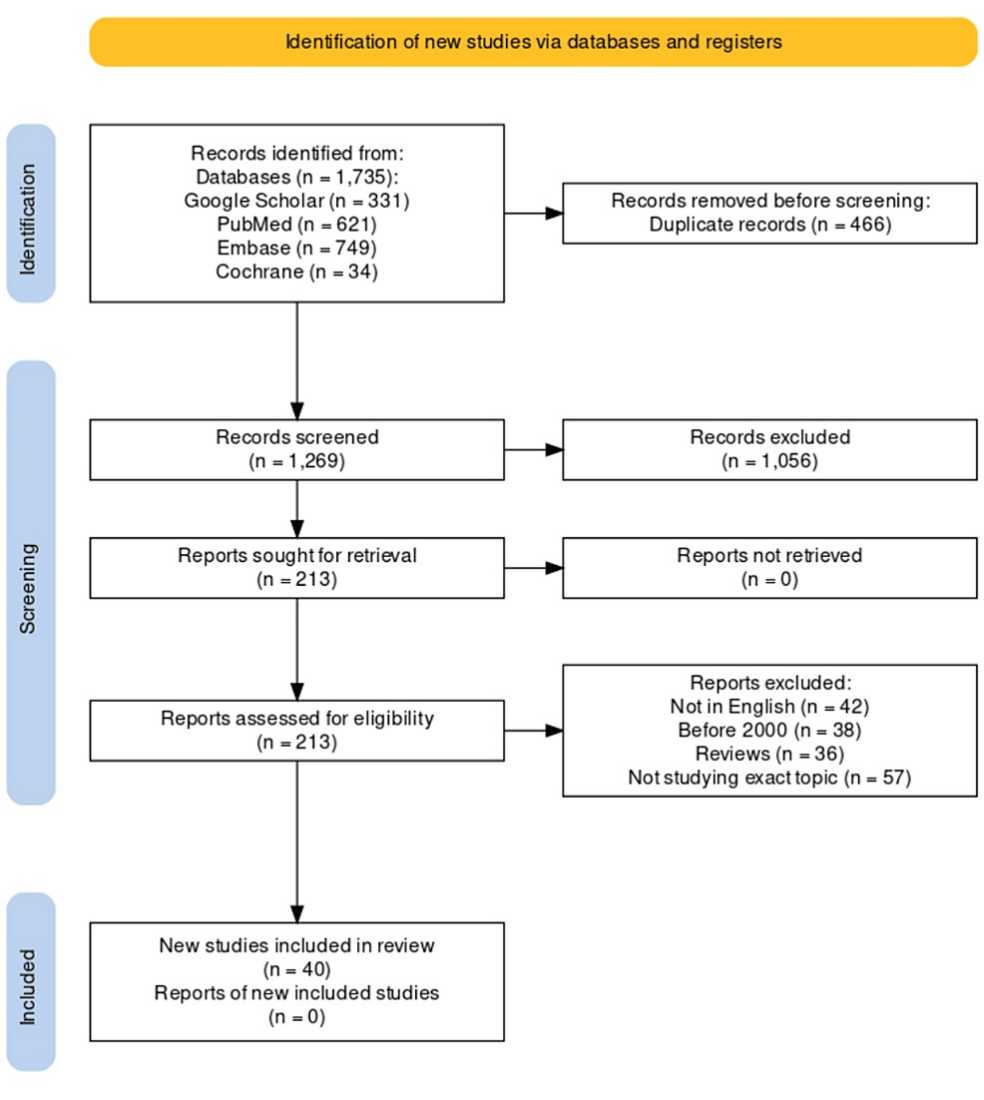
PRISMA flowchart

The thorough literature search across the four electronic databases indicated previously turned up 1735 relevant articles. The two reviewers in charge of completing the literature search found duplicates and eliminated the 466 articles. After carefully reviewing the remaining articles' titles and abstracts and content and conducting the eligibility assessment, only 40 studies met the requirements for the review [17,18,20,28-64]. The PRISMA literature search criteria are presented in Figure 1.

Characteristics of Included Studies

The data analyzed in the current study was obtained from 40 papers, as summarized in Tables 1-2. Of the 40 studies, 15 were retrospective case series and case reports, four were retrospective cohort studies, 16 were reviews, and the remaining a meta-analysis, protocol, research, and cross-sectional studies, one each. The studies were conducted across various countries, including Brazil, Turkey, Portugal, China, Spain, Mexico, Japan, Canada, Norway, the Netherlands, and Taiwan, constituting data of pregnant women with MG collected from 1967 to 2022. As reported in Table 1, there was a total of 1322 women with MG, with 1604 pregnancies recorded. The mean maternal age ranged from 25.8 to 48.1 years, while the gestational age ranged from 36.7 to 39.4 weeks (see Table 1).

**Table 1:** Patient and sample characteristics MG: myasthenia gravis, ns: not specified, na: not applicable

Bibliographic details		Patient characteristics					
Author, year	Setting, country	Sample size (n)	Maternal age (years)	Gestational age (mean weeks)	Improvement	Deterioration or exacerbation	No change
Almeida et al., 2010 [28]	Hospital de Santo Anto´ nio, Porto, Portugal	15 patients	28 ± 4.3	-	-	4 exacerbations	8
Braga et al., 2016 [18]	Centro Hospitalar do Porto, Oporto, Portugal	25 patients and 30 pregnancies	32.4 ± 4.1	38.2	1	43.3% and 46.4% occurred at postpartum	ns
Ducci et al., 2017 [30]	Brazil	21 patients with 30 pregnancies	25.8 ± 5.2	38	9 (30%) MG scores (p = 0.012) before pregnancy	15 (50%) P = 0.028	6 (20%) longer MG duration (p = 0.026)
Gamez et al., 2017 [31]	Spain	5 pregnant MG patients	36.4 ± 5.8	38 ± 5	-	-	5
Santos et al., 2018 [32]	Portugal	17 women with 25 pregnancies among 13 women	26.2	-	-	1 exacerbation	-
Shi and Zeng, 2018 [33]	Hospital of Wenzhou Medical University, China	8 MG pregnancies	27.5	38.13	-	3	-
Tanacan et al., 2019 [17]	Hacettepe University Hospital, Turkey	27 pregnancies in 12 patients	29.3	36.7	7	11 deteriorations, 25.9% exacerbations	9
Téllez-Zenteno et al., 2004 [34]	National Institute of Perinatology, Mexico City, Mexico	18 patients	27.4 ± 4.0	37.5 ± 3.0	2 (11%)	7 (39%)	9 (50%)
Tsurane et al., 2019 [35]	Japan	6 pregnant MG patients	32.3	38 ± 3	2	1 exacerbation	-
Alharbi et al. 2021 [36]	Prosserman Family Neuromuscular Clinic, Canada	20 women with 28 pregnancies	29.7 ± 5.7	-	-	50%	50%
Boldingh et al. 2016 [37]	Norway and Netherlands	246 women	48.1 (15.5)	-	-	30% to 40%	60% to 70%
Su et al. 2022 [38]	na	734 pregnancies	-	-	51	193	490
Qi et al., 2012 [39]	Peking Union Medical College Hospital, China	38, 683 women, 9 patients with MG	-	-	1	3 terminations	6
Zhou et al., 2022 [29]	Xiangya hospitals, China	37 MG pregnancy among 33 women	32.4	-	-	5.4% and 38.9%, exacerbation in 3^rd^ trimester	-
Hoff et al., 2003 [40]	Norway	127 births in 79 MG women	29	39.4	na	40.9%	-
Hoff et al., 2007 [41]	Norway	73 MG mothers with 135 births	29	-	-	13 (10%) deteriorations	-
Wen et al., 2009 [42]	Taiwan	163 MG mothers	27	38	na	-	-
Cheng et al., 2007 [43]	Taiwan	13 pregnancies in 12 MG women	31.6	37.2 ± 2	-	38.5% exacerbations and 10 deteriorations	­-

The Course of MG During Pregnancy and Complications

Across the 16 studies exploring the course of MG during pregnancy, there were 401 (30.4%) exacerbations of the conditions, while deteriorations were 35.05% with 8.753% improvement (Table 1). While most women delivered normally, through vaginal delivery, a significant number of cases required a caesarian section for delivery. Among the studies reporting complications for pregnant women, there was a 10.99% miscarriage rate, myasthenic crisis (6.67%), 11.4% spontaneous abortions [28], preterm deliveries, and pregnancies with preterm premature rupture of membranes (PPROM) reported. Infant-related outcomes reported include transient neonatal MG (TNMG), which were 17 (17.17%) cases of transient MG among the studies reporting the outcome with mortalities cases, Down syndrome, deformity, icterus, and erythroblastosis being reported among infants (see Table 2 for study characteristics) [29].

**Table 2:** Study characteristics MG: myasthenia gravis, RS: retrospective case series, RCS: retrospective cohort study, CS: cesarean section, OC: obstetric complications, PPROM: preterm premature rupture of membranes, NMG: newborn/neonatal myasthenia gravis, TNMG: transient myasthenia gravis, VD: vaginal delivery, IVIg: intravenous immunoglobulin, MuSK: muscle-specific kinase: ELA: epidural labor analgesia, MA: meta-analysis, AChr: acetylcholine receptor, SGA: smaller gestational age, na: not applicable, GDM: gestational diabetes mellitus, SA: spontaneous abortions, PROM: premature rupture of amniotic membranes, LBW: low birth weight

Bibliographic details			Study characteristics					
Study ID	Study design	Study period	Study objectives	MG treatment medication	Mode of delivery	Associated complications	Infant outcomes	MG and pregnancy
Almeida et al., 2010 [28]	RS	1985 to 2009	Analyze the peripartum problems and anesthesia management for MG patients	Pyridostigmine, IVIg, and prednisolone	CS (8), VD (1)	Myasthenic crisis in one patient	ns	Although the disease typically worsens, MG can slightly interfere with pregnancy and delivery
Braga et al., 2016 [18]	RS	2005 to 2013	Evaluating clinical course during pregnancy and neonatal outcomes	Pyridostigmine (80%), corticosteroids (43.3%), and IVIg (40%)	CS (64.3%)	Miscarriage rate (6.7%) with 28 newborn deliveries	2 TNMG in 28 newborns	High risk of clinical MG worsening in the mother
Ducci et al., 2017 [30]	RS	1990 to 2015	Outcome and impact of pregnancy in women with MG	Prednisone, pyridostigmine, azathioprine, and IVIg	CS (66.7%), VD (73.3%)	OC in 20 pregnancies with PPROM (25.8%), abortion (11.4%), and fatal death (2.9%)	12.9% TNMG in 31 infants	PPROM and cesarean births are more common in pregnant MG patients
Gamez et al., 2017 [31]	RS	2013 to 2014	Efficacy of IVIg as a single therapy for pregnant MG women	IVIg	CS (3), VD (2)	-	-	IVIg monotherapy during pregnancy in MG patients may be beneficial
Santos et al., 2018 [32]	RS	ns	Pregnancy's impact on pregnancy outcomes and how MuSK-MG progresses	Pyridostigmine (4), Azathioprine (1), and IVIg (1)	CS (6), VD-(18)	3 miscarriages	1 neonatal MG	ns
Shi and Zeng, 2018 [33]	RS	2004 to 2012	Management of MG in pregnancy	Prednisone (3), prednisone + pyridostigmine (2), and pyridostigmine (2)	CS (3), VD (5)	1 preterm delivery	1 newborn developed TNMG	Women with unstable MG should delay pregnancy to reduce the chance of MG exacerbation and unfavorable consequences on the fetus
Tanacan et al., 2019 [17]	RS	Jan 1, 2010, to Dec 31, 2017	Pregnancy management for MG patients	Pyridostigmine, prednisolone, and IVIg	CS (78.3%), VD (21.7%)	Four (14.8%) miscarriages, 3 (11.1%) preterm births, and 4 (14.8%) PPROM	6 (26%) TNMG	A multidisciplinary approach is required for MG management in women
Téllez-Zenteno et al., 2004 [34]	RS	Jan 1, 1996 to Dec 31, 2003	ns	Pyridostigmine (13), pyridostigmine plus steroids (1), and azathioprine + steroids (1)	VD (9), CS (8)	1 fatal loss	1 TNMG in 17 infants	Variable clinical outcomes for MG during pregnancy
Tsurane et al., 2019 [35]	Protocol study	Mar 2016 to Nov 2017	Validity of ELA about the seriousness of MG	Prednisolone	VD	-	ns	Women with MG can safely have spontaneous or surgical VD
Alharbi et al. 2021 [36]	RS	2001 to 2019	Reviewing local knowledge of MG, pregnancy, and results	Azathioprine 10 (35.7%), prednisone 13 (46%), IVIg 4 (14%)	CS (29%), VD (71%)	2 deliveries, 2 (7%) premature births	2 NMG	MG worsened in a high proportion of patients
Boldingh et al., 2016 [37]	Cross-sectional study	ns	Possibility of MG developing clinically both during pregnancy and after delivery	Prednisone, IVIg	VD	ns	ns	High risk of MG symptoms in the postpartum period
Su et al., 2022 [38]	MA	na	The relationships between clinical variables and MG's pregnancy-related outcomes	Anticholinesterase	CS (OR 0.39, 95% CI 0.05-3.21)	Preterm delivery (OR 3.06 95% CI 0.97-9.69), ocular MG (OR 0.54, 95% CI 0.07-4.41)	ns	Pregnancy-related MG's overall proportion of deterioration and improvement was 0.36 (95% CI 0.25-0.40) and 0.28 (95% CI 0.17-0.40), respectively
Qi et al., 2012 [39]	RS	1983 to 2010	Pregnancy and MG interaction and management	Thymectomy	CS (5), VD (2)	Preterm delivery	3 SGA	Three cases of thymectomy stayed stable, while one got worse during pregnancy
Zhou et al., 2022 [29]	RCS	2012 to 2022	Adverse pregnancy outcomes and postpartum aggravation in Asian MG women	ns	VD, CS	PROMS, GDM, and thyroid complications	TNMG and hyperbilirubinemia (24.3%)	While most MG patients have normal pregnancies, there is an increased incidence of maternal and fetal problems
Hoff et al., 2003 [40]	RCS	1967 to 2000	Maternal MG's impact on birth and the newborn	Thymectomy, Bromocriptine pyridostigmine	CS (33), VD (11)	PPROM, postpartum bleeding, and birth obstruction	NMG, 3 mortalities, down syndrome, deformity, icterus, and erythroblastosis	Regarding the reference group, MG women have a greater risk of PPROM (5.5% vs. 1.7%, p 0.001) and higher delivery problems (40.9% vs. 32.9%, p 0.05). Increased rates of delivery interventions (33.9% vs. 20.0%, p 0.001) and CS (17.3% vs. 8.6%, p 0.001) were observed
Hoff et al., 2007 [41]	RCS	1967 to 2004	Causes of the higher complication rate in MG women	Thymectomy	CS (13%), VD	PROM, bleeding >1500 ml, SA	NMG neonatal distress	NMG is linked to fetal discomfort during birth (P = 0.05). MG women use medication during pregnancy (P 14 0.001), thymectomies (P 14 0.007), and undergo elective CS (P 14 0.009). Thymectomy may protect against NMG
Wen et al., 2009 [42]	RCS	2001 to 2003	Risk of adverse pregnancy outcome in MG women	Thymectomy	CS (44.8%, vs. 37.4%)	Preterm births (8.1%)	LBW, SGA	LBW, preterm birth, SGA babies, and cesarean delivery rates for moms with MG were 1.19 (95% CI = 0.60-2.38), 1.00 (95% CI = 0.54-1.87), 1.30 (8.2-20.4), and 1.33 (95% CI = 0.94-1.88), respectively, compared to mothers who weren't affected
Cheng et al., 2007 [43]	RS	1997 to 2005	Neonatal outcomes of expectant MG mothers	ns	VD (2), CS (10), elective CS (33%)	Upper airway tract infection	TNMG and maternal anti-AChR titer, congenital anomaly (14.2%)	Except for one patient who declined in the first trimester and had an upper respiratory tract illness, all MG patients progressed postpartum
Kühnert et al., 2021 [44]	Review	ns	Section Maternal Disease Guidelines for MG in pregnancy	Pyridostigmine, IVIg, azathioprine	CS	ns	NMG	Stable MG in medical adjusted before pregnancy and high risk of MG exacerbation during the acute postpartum period
Gilhus, 2023 [45]	Review	na	Pregnancy-related MG treatment considerations	Prednisolone, azathioprine	VD, CS	Miscarriage, SA (22%)	Neonatal MG, fetal AChR, inactivation syndrome	MG medications are secure and do not raise the chance of deformities
Gilhus, 2020 [46]	Review	na	MG can affect pregnancy and a child's development	Pyridostigmine, prednisolone, azathioprine	VD, CS	SA	Neonatal MG	Like the non-MG population, pregnancy and childbirth have an equal incidence of complications. It is advised to give birth vaginally
Binks et al., 2016 [47]	Review	2007	Clinical-immunological update	IVIg, pyridostigmine, corticosteroids	ns	ns	ns	Understanding MG requires more research, and treating aged people presents diagnostic and therapeutic challenges
Neykoya et al., 2022 [48]	Review	ns	Interrelations of symptoms, clinical outcomes and treatment regimens in MG pregnant women	Pyridostigmine, azithromycin	VD, CS	Miscarriage, malformations	-	Co-infection with COVID-19 and MG during pregnancy does not worsen either of those illnesses
Banner et al., 2022 [49]	Review and case series	ns	Prenatal care and attention to fetal and neonatal problems in mothers with MG	Pyridostigmine, corticosteroids, IVIg, cyclosporine	VD, CS	-	NMG	For women with MG, intensive maternal and pregnancy monitoring by a multidisciplinary team can enhance pregnancy outcomes
Roche and Bouhour, 2021 [50]	Review	ns	Reducing the effects of MG during pregnancy and on the unborn child and preventing myasthenia crises in the postpartum	Pyridostigmine prednisone, azathioprine	VD	Malformations, gestational diabetes PROM	TNMG	The risk of MG aggravation postpartum (about 30%) is reduced under MG control. TNMG occurs regardless of maternal illness management
Cimpoca-Raptis et al., 2021 [51]	Review	ns	Prenatal care and to draw attention to fetal and neonatal problems in moms with MG	Pyridostigmine, azathioprine, IVIg, corticosteroids	VD, CS	SA miscarriage	TNMG	Less than 1% of pregnant women have fetal arthrogryposis, while 10% to 20% get TNMG. Although the course of MG might be uncertain at times, many pregnant women remain stable
Varner, 2013 [52]	Review	ns	Pregnant women with MG	Pyridostigmine, azathioprine, IVIg, corticosteroids	VD, CS	Spontaneous preterm delivery miscarriage	NMG	The impact of myasthenia during pregnancy varies significantly from woman to woman and from pregnancy to pregnancy within the same woman
Waters, 2019 [53]	Review	ns	Treatment of MG during Pregnancy	Pyridostigmine, prednisone, azathioprine, IVIg	CS, VD	Miscarriage spontaneous preterm delivery	TNMG	40% of MG expectant mothers have increased symptoms, and 20% need ventilator support. There is a 10% to 20% chance of TNMG in children born to MG moms
Bansal et al., 2018 [54]	Review	ns	Pregnancy-related MG treatment	Azathioprine, mycophenolate, pyridostigmine	VD, CS	PROM SA	NMG	A healthy pregnancy requires meticulous planning, a multidisciplinary team approach, and careful attention to both the mother's and fetus' well-being
Grover and Sripathi, 2020 [55]	Review	ns	-	Pyridostigmine, azathioprine, prednisone	CS, VD	PROM and preterm delivery	TNMG	-
Alfaro-Paredes et al., 2022 [56]	Review	ns	Association between MG and pregnancy, along with its strategy	Azathioprine, pyridostigmine, prednisone	VD, CS	SA premature delivery	TNMG	A multidisciplinary approach should be used when planning a pregnancy in myasthenic individuals
Roth et al., 2015 [57]	Clinical report	ns	Analyzing the illness process and how it affects the prenatal, labor, and delivery periods	Pyridostigmine, prednisone, azathioprine	CS, VD	Prematurity	NMG	-
Ciafalon and Janice, 2004 [58]	Review	ns	Management of the pregnant woman and the neonate with MG	Azathioprine, pyridostigmine, prednisone	VD, CS	SA	TNMG	It is possible to successfully manage MG during pregnancy and after delivery, but this involves cooperation between the obstetrician, the neurologist, and a knowledgeable patient
Vu et al., 2021 [59]	Case report	ns	Eculizumab medication before, during, and after pregnancy for successful pregnancy in a woman with treatment-refractory MG	Pyridostigmine, prednisone, azathioprine	VD, CS	preterm delivery, PROM miscarriage	NMG	The patient on five-year eculizumab treatment remained neurologically stable
Massey and De Jesus-Acosta, 2014 [60]	Review	ns	Incidence of MG in women of childbearing third decade	Pyridostigmine, prednisone, IVIg, azathioprine, cyclosporine	CS, VD	SA, prematurity	TNMG	Individualized treatment plans must consider the MG severity, the distribution of weakness, any concurrent disorders, and the welfare of the fetus
Shimizu and Kitagawa, 2016 [61]	Review	na	Pregnancy effect and management of MG in pregnancy	Prednisone, azathioprine, methotrexate, pyridostigmine, cyclosporine	VD, CS	SA, premature birth, and pre-eclampsia	TNMG	Exacerbations happen throughout the first trimester and the first three months after giving birth. About 10-30% of children born to MG mothers have TNMG
Hamel and Ciafaloni, 2018 [62]	Review	na	Management of MG in pregnant women	Azathioprine, corticosteroids, rituximab, thymectomy, pyridostigmine, cyclosporine	CS, VD	Premature delivery, SA	TNMG	Neurologists, obstetricians, and anesthesiologists should support MG patients during pregnancy and postpartum. Newborns of MG mothers are at risk of TNM. Pregnancy outcome is favorable in MG women who receive treatment
Benjilany and Kouach, 2021 [63]	Case report	na	MG in pregnancy	Corticosteroid, thymectomy	VD, CS	SA, PROM	NMG	Maternal myasthenic decompensation and NMG concerns necessitate strict and interdisciplinary management of MG patients
Norwood et al. 2014 [64]	Research study	na	MG in pregnancy	Corticosteroid, pyridostigmine, azathioprine, IVIg	VD, CS	ns	TNMG	TNMG is a concern for newborns born to myasthenic mothers. VD is encouraged, and multidisciplinary management for severe MG
Lee et al., 2017 [20]	Case report	na	TNMG due to MG in woman	Pyridostigmine, IVIg	CS	Ptosis and MuSK positive	TNMG, respiratory failure, and hypotonic	Pregnant women with suspected ocular MG must have MuSK antibody testing to detect TNMG

Recommended Medications for Women With MG

The majority of women across the studies were administered with treatments mainly comprising of drugs such as prednisone, corticosteroid, pyridostigmine, rituximab, cyclosporine, azathioprine, methotrexate, and IVIg (see Table 2). The percentage of women who underwent a thymectomy before becoming pregnant ranged from 16% to 100% [31]. Most pregnant women were treated with anticholinesterase drugs across studies, although a sizable proportion also required prednisone, IVIg, and azathioprine [18,19,28,30]. Table 3 below summarizes the common medications for pregnant women with MG [14].

**Table 3:** Summary of recommended medication for MG during pregnancy IVIg: intravenous immunoglobulin, AEs: adverse effects

Drug/treatment	Administered as	Dosage administration	Expected time for action onset	Common side effects
Prednisone	Induction therapy	10 mg daily increased to 60-100 mg for 2-4 weeks	2-4 weeks	Fluid retention, neuropsychiatric, hyperglycemia, hypertension, and density loss
Thymectomy	First-line treatment	-	6-12 months	-
Pyridostigmine	Induction therapy	60 to 120 mg for 3-8 hours/day	≤30min	Nausea and vomiting, diarrhea, and loose stool
IVIg	Secondary therapy	2 g/kg for 2-5 days	1-2 weeks	Nephrotoxic, urticaria, headache, and thromboembolic events
Azathioprine	Secondary treatment	50 mg doubled every 2-4 weeks	12-18 months	Nausea and vomiting, flu-like illness, and leukopenia
Cyclosporine	Secondary treatment	100 mg twice daily	1-3 months	Hypertension, infection, tremor, hyperplasia, nephrotoxicity, and neoplasia
Methotrexate	Third-line treatment	10 mg weekly	-	Hepatotoxicity, infection, fibrosis, and neoplasia
Rituximab	Fourth	375 mg/mm^2^ a week for 4 weeks	1-3 months	Leukopenia, chills, headache, nausea, thrombocytopenia, and hypotension
Cyclophosphamide	Fifth-line therapy	0.5-1 g/m^2^	6-12 months	Hemorrhagic cystitis, infections, nausea, alopecia, and bone marrow suppression
Eculizumab	Fifth-line	900 mg weekly for 4 weeks	2-4 weeks	Meningococcal infections and mild infusion AEs

Quality and Risk of Bias Assessment

**Figure 2:**
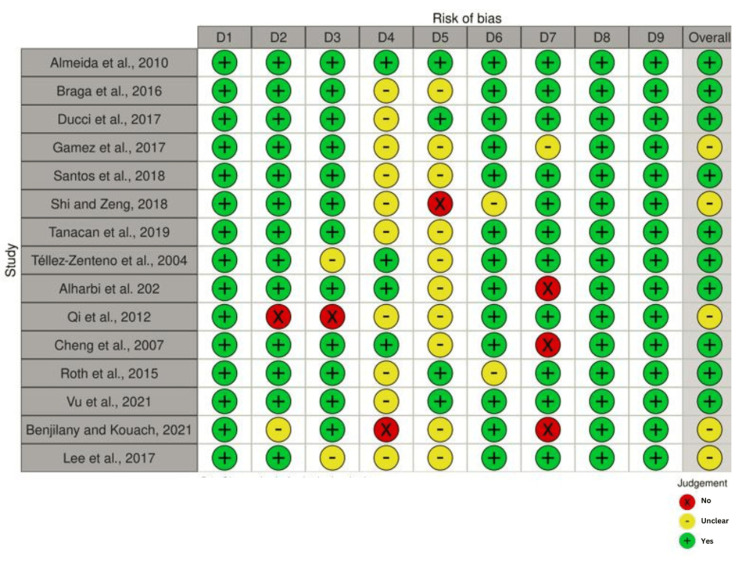
Traffic light plot for 15 case series studies Studies [17, 18, 20, 28, 30-33, 36, 39, 43, 57, 59, 63]. D1: clear criteria for inclusion in the case series, D2: condition measured in a standard reliable way for all participants, D3: valid methods used for identification of the condition of all participants, D4: consecutive inclusion, D5: complete inclusion, D6: clear reporting of demographics of the participants, D7: clear reporting of clinical information, D8: outcomes or follow-up results clearly recorded, D9: clear reporting of presenting site demographic information

**Figure 3:**
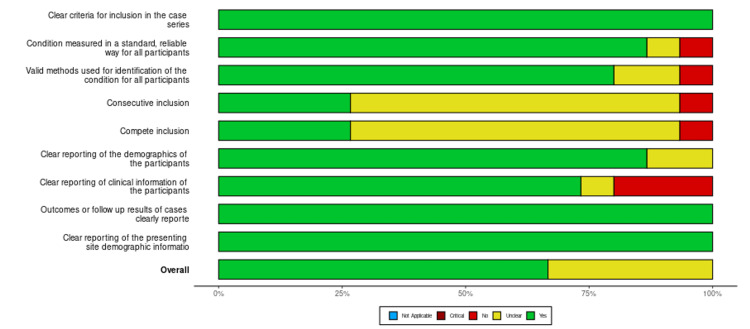
Risk of bias summary for case series

**Figure 4:**
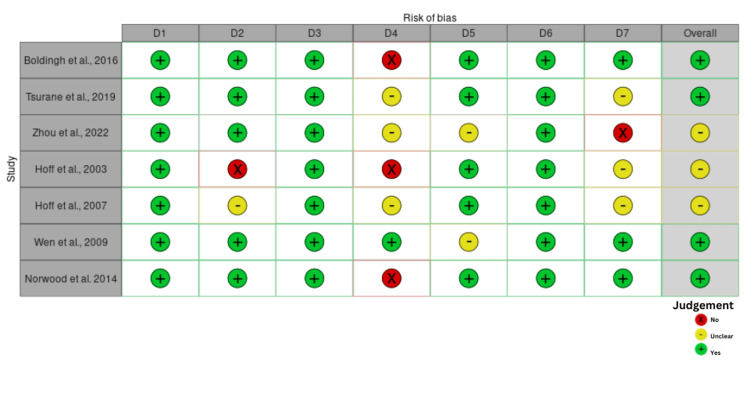
Risk of bias for cohort studies Studies [29, 35, 37, 40-42, 64]. D1: groups similar or recruited from the same population, D2: exposures measured similarly to assign people, D3: exposure measured in a valid and reliable way, D4: confounding factors, D5: outcomes measured in a valid and reliable way, D6: follow-up time reported and sufficient, D7: appropriate statistical analysis use

**Figure 5:**
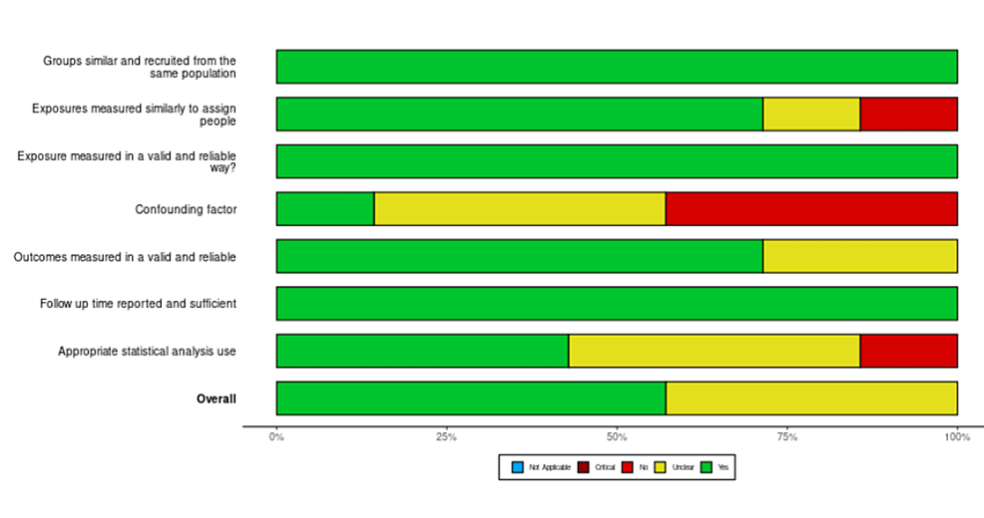
Risk of bias summary for cohort studies

Based on the JBI critical tool for methodological quality of retrospective case series, 10 were of low risk of bias, while the remaining five were of moderate quality [27]. There was no case study of poor quality. The JBI tool for retrospective cohort studies gave four studies of high quality and five of moderate quality (Figures 2-5).

Discussion

MG in women of childbearing age is a critical condition warranting careful consideration and assessment, especially during pregnancy. As an uncommon neuromuscular condition, MG significantly impacts pregnant women, which can be culminated with exacerbation of the condition during pregnancy. The current study explores the impact of MG in pregnancy and the association between the two. The results of the present study demonstrate a wide range of risks associated with MG during pregnancy, in which 30.4% of exacerbations were recorded among pregnant women, with a corresponding deterioration of 35.05%. The condition only stabilized or improved among 8.57% of the women. The implication of these outcomes reveals the unpredictability of the course of MG in pregnant women. Pregnancy, therefore, can affect each woman and successive pregnancies in the same woman differently in terms of how it impacts their MG. This unpredictability is demonstrated by the fact that while in some cases there is no change or the MG stabilizes, in other cases, pregnant women experience improvements or deterioration and exacerbation of the condition.

Prior studies also showed consistent results, with a sizeable portion of women (29%) experiencing improvement. Contrarily, there was no change, or MG stabilized in 31% of cases, with 40% of pregnant women experiencing deteriorating conditions [65]. Ferrero et al. reiterated that symptoms worsened for 41% of pregnant women with MG, compared to 30% who exhibited no change and 29% who went into remission. Similarly, the percentage of MG exacerbations during pregnancy can range from 0% to 60% in an individual series [31,66]. The 30.4% of MG symptoms exacerbation in the present study falls in range with the overall cited risk of between 30% and 45%, consistent with a previous systematic review whose symptoms worsening was recorded at 33.8% [19,34,49]. The majority of this worsening, according to two of the included studies, mainly occurred during the postpartum period (43.3% to 46.4%) and in the third trimester (5.4% to 38.9%) in pregnant women [18,40]. However, Chaudhry et al. observed that typical immunosuppressive changes in late pregnancy have been linked to symptoms improving in the second and third trimesters. In contrast, symptoms will likely worsen in the first trimester or the days following delivery [10].

Following the course of MG in pregnancy, the likelihood of maternal mortality likely happening in the first year of the diagnosis of MG, and the fact that is less likely to happen seven years later [19], several previous studies have recommended pregnancy delay for at least two years. By doing so, especially in women with unstable MG, the risk of MG exacerbation lessens, thus lowering the associated risks to the fetus [19,33]. On the other hand, the present study observed that myasthenic crises were a critical and potentially fatal MG complication occurring at a rate of 6.67% and were reported in one pregnant woman [30]. Likewise, Banner et al. found that myasthenic crisis occurred in 6.4% of pregnant women during pregnancy and 8.2% during postpartum [49]. Other reported complications include preterm deliveries, spontaneous abortions, and PPROM. Abortions were reported among 11.4% of pregnant women with MG [17,18,32,45,46]. In contrast to the 3% risk in the general population, PPROM appears more common in women with MG, which affects 6.7% of all pregnancies [17,28,67].

There were reported instances of the impact of MG on infants born to mothers with MG. Incidences of babies with fetal growth restriction (FGR) and TNMG were common among these mothers [39,42]. The odds ratio of low birth weight (LBW) and FGR among the neonates were higher than 1 [42]. Similarly, Banner et al. observed that 14.1% of infants born to women with MG have LBW less than the gestational age [49]. Our study reports a 17.17% rate of TNMG among infants born to mothers with MG. Our findings align with earlier studies that noted the potential for TNMG to manifest in babies. TNMG can be explained as since immunoglobulin G antibodies are delivered through the placenta in the second and third trimesters, TNMG in newborns born to MG moms happens in 10% to 20% of cases [68]. Close monitoring is, therefore, necessary because the baby frequently displays indications of TNMG two to four days after birth, including respiratory problems, muscle weakness, a feeble scream, poor sucking, and ptosis [5,19]. Eventually, the condition reverses as the mother's antibodies deteriorate of their volition after two to three weeks [19].

Among deliveries, some studies find that less than 50% were done via vaginal deliveries among pregnant women with MG, while the rest were cesarean sections [29,35,37,40-42,64]. Contrary, another review found that vaginal delivery was the most common mode, with 56.3% of pregnant women giving birth through spontaneous vaginal deliveries [49]. Nonetheless, there is some worry that for mothers experiencing symptoms of MG at the time of delivery, the voluntary striated muscles required for vigorous pushing may be reduced by MG exacerbation and further damaged by excessive maternal effort, leading to the myasthenic crisis [68]. An aided second stage of labor may reduce the requirement for maternal action and mitigate this risk in women experiencing exacerbation symptoms at birth. Since the uterus is formed of smooth muscle and is unaffected by ACh receptor antibodies, vaginal delivery is advised for MG patients [68]. However, due to the involvement of striated muscles during the second stage and the potential impact of the ACh receptor antibody on these muscles, assisted or vacuum extraction may be necessary for VD [68,69]. In the event of cesarean section, epidural anesthesia is recommended for use during labor and delivery since opioids and neuromuscular drugs can amplify the effects of ACh receptor antibodies on the neuromuscular junction [68]. The mode of delivery is an important consideration. Vaginal delivery is possible if the mother's respiratory muscles are strong enough to tolerate the increased effort of labor. However, in cases of severe myasthenia or respiratory compromise, a cesarean section may be recommended to avoid excessive stress on the respiratory system. The decision is based on the mother's clinical condition, fetal well-being, and obstetric factors [29,35,37,40-42,64].

Pharmacologic therapy concentrates on raising ACh levels and lowering the production of autoantibodies and is the mainstay of MG treatment and management [14]. However, the pharmacologic course of treatment during pregnancy may need to be modified depending on the severity or escalation of the condition [69]. Acetylcholine esterase inhibitors such as pyridostigmine, IVIg, azathioprine, steroids, and prednisolone are often used in the therapy interventions documented in this study [14]. Although there is limited data and information on acetylcholine esterase inhibitors during pregnancy, none indicates an increased risk of deformity or other unfavorable pregnancy outcomes [2,14]. Watching for a myasthenic crisis is very important. Pregnancy places additional stress on the body, and myasthenic mothers may experience myasthenic crises during pregnancy or postpartum. As the crisis can cause severe muscle weakness, respiratory failure, and bulbar weakness, prompt recognition and treatment are crucial. Management involves ensuring adequate respiratory support and administering IVIg or plasmapheresis to stabilize the condition [35,37,40-42,64]. The role of thymectomy is debatable but it is a definitive treatment for MG. Thymectomy can be performed before or during pregnancy, and the decision should be individualized based on the patient's condition. Studies suggest that thymectomy can lead to disease remission or improvement in a significant number of patients, potentially reducing the need for long-term immunosuppressive medications during pregnancy. However, the timing of thymectomy should be carefully considered to optimize the benefits and minimize risks to both the mother and the fetus [39].

Limitations

The majority of the included studies were small and did not employ a comparison design, limiting the current investigation due to its small sample sizes and inclusion of low-quality evidence. Case studies and reviews are especially prone to convenience sampling and selection bias, unlike randomized controlled trials. They might not accurately represent the general population of pregnant women with MG despite gathering information from various parts of the globe. Additionally, the lack of a comparison group in most of the included studies reduces internal validity and forces descriptive reporting of data rather than statistically relevant analysis of effect measures across the studies. Besides, most studies examined tracked pregnancies rather than particular individuals, which increased the probability that some data were unreliable.

## Conclusions

MG is a condition that has a high risk of complications, especially when a woman is pregnant. Severe illnesses that may even be life-threatening, such as respiratory insufficiency that puts both the expectant mother and the unborn child in danger, may occur, especially due to generalized weakness. Compared to the general population, women with MG are more likely to need an assisted vaginal birth or a cesarean section. With the exception of PPROM, MG does not appear to raise the incidence of adverse outcomes significantly. Regardless of antenatal risk factors or maternal status, neonates should be watched for symptoms of TNMG. Additionally, given that this condition mainly affects fertile women, it is imperative to be knowledgeable about MG and the interdisciplinary diagnostic and therapeutic care it requires.
